# Supplementary material for: Coaching styles and sports motivation in athletes with and without Intellectual Impairments
Source: PLoS One. 2023 Dec 22;18(12):e0296164. doi: 10.1371/journal.pone.0296164 (PMC10745216; doi:10.1371/journal.pone.0296164)
Supplement: S1 Checklist — (DOCX) [file pone.0296164.s001.docx]

STROBE Statement—checklist of items that should be included in reports of observational studies

|  | Item No. | Recommendation | Page  No. | Relevant text from manuscript |
| --- | --- | --- | --- | --- |
| **Title and abstract** | 1 | (*a*) Indicate the study’s design with a commonly used term in the title or the abstract | 1 | Coaching styles and sports motivation in athletes with and without Intellectual Impairments (cross-sectional study) |
|  |  | (*b*) Provide in the abstract an informative and balanced summary of what was done and what was found | 2 | The cognitive limitations……..of people with II. |
| Introduction | | | |  |
| Background/rationale | 2 | Explain the scientific background and rationale for the investigation being reported | 4-7 | According to the convention of the rights of individuals with disabilities……the exploration of the athletes’ motivation and progression from different perspectives will give us the opportunity to deeper explore the coach-athlete relationship in sports settings |
| Objectives | 3 | State specific objectives, including any prespecified hypotheses | 7 | it aims to examine if: 1) there are differences in…….that these predictors differ between the two groups (II and non-II). |
| Methods | | | |  |
| Study design | 4 | Present key elements of study design early in the paper | 7-10 | Participants, Procedures, Measurement, and Statistical Analysis |
| Setting | 5 | Describe the setting, locations, and relevant dates, including periods of recruitment, exposure, follow-up, and data collection | 7-8 | Recruitment of coaches of athletes with and without II athletes was done through sports organisations, recreational centers and sports clubs via phone calls and e-mails (from January until May of 2021). The authors did not have access to information that could identify individual participants during or after data collection. |
| Participants | 6 | (*a*) *Cohort study*—Give the eligibility criteria, and the sources and methods of selection of participants. Describe methods of follow-up  *Case-control study*—Give the eligibility criteria, and the sources and methods of case ascertainment and control selection. Give the rationale for the choice of cases and controls  *Cross-sectional study*—Give the eligibility criteria, and the sources and methods of selection of participants | 7-8 | Recruitment of coaches of athletes with and without II athletes was done through sports organisations, recreational centers and sports clubs via phone calls and e-mails.  We included coaches who were fluent in English, had at least one year of coaching experience, and their athletes were adolescents or adults (aged 12 or above)  Their athletes should be categorized in the ‘participation’ or ‘performance’ stage of sports development (focus on sports skills development with experience in competitive events)  Athletes with II must meet the criteria for diagnosis of II as set by the British Psychological Society |
|  |  | (*b*) *Cohort study*—For matched studies, give matching criteria and number of exposed and unexposed  *Case-control study*—For matched studies, give matching criteria and the number of controls per case | 8 | Two hundred and sixty-six coaches with coaching experience in different sports (e.g., athletics, gymnastics, basketball, football etc.) consented to participation (45.9% coaches of athletes with II). Their average age was 40.88 (SD = 16.07, range 17 to 81 years old) and 58.6% of them were males. |
| Variables | 7 | Clearly define all outcomes, exposures, predictors, potential confounders, and effect modifiers. Give diagnostic criteria, if applicable | 8-9 | Coaches of athletes with and without II completed questionnaires……and showed a strong factor structure, internal consistency, and validity [38]. |
| Data sources/ measurement | 8* | For each variable of interest, give sources of data and details of methods of assessment (measurement). Describe comparability of assessment methods if there is more than one group | *8-9* | Coaches of athletes with and without II completed questionnaires …….and showed a strong factor structure, internal consistency, and validity [38]. |
| Bias | 9 | Describe any efforts to address potential sources of bias | 8-9 | The authors did not have access to information that could identify individual participants during or after data collection. Standardised, valid and reliable measurements |
| Study size | 10 | Explain how the study size was arrived at |  | The study size was calculated by using the G power. For the II group however, we used all the potential participants nationally (UK). |

Continued on next page

| Quantitative variables | 11 | Explain how quantitative variables were handled in the analyses. If applicable, describe which groupings were chosen and why | 8-10 | Total 6 variables |
| --- | --- | --- | --- | --- |
| Statistical methods | 12 | (*a*) Describe all statistical methods, including those used to control for confounding | 10 | Shapiro-Wilk test,a rank MANOVA, Additive Nonparametric Regressions (Generalized additive model), |
|  |  | (*b*) Describe any methods used to examine subgroups and interactions | 10 | rank MANOVA, Additive Nonparametric Regressions |
|  |  | (*c*) Explain how missing data were addressed | 10 | We did not have missing data due to the study design (online) |
|  |  | (*d*) *Cohort study*—If applicable, explain how loss to follow-up was addressed  *Case-control study*—If applicable, explain how matching of cases and controls was addressed  *Cross-sectional study*—If applicable, describe analytical methods taking account of sampling strategy | - | - |
|  |  | (*e*) Describe any sensitivity analyses | - | We did not provide a sensitivity analysis |
| Results | | | | |
| Participants | 13* | (a) Report numbers of individuals at each stage of study—eg numbers potentially eligible, examined for eligibility, confirmed eligible, included in the study, completing follow-up, and analysed | 8 | Two hundred and sixty-six coaches with coaching experience in different sports (e.g., athletics, gymnastics, basketball, football etc.) consented to participation |
|  |  | (b) Give reasons for non-participation at each stage | - | - |
|  |  | (c) Consider use of a flow diagram | - | - |
| Descriptive data | 14* | (a) Give characteristics of study participants (eg demographic, clinical, social) and information on exposures and potential confounders | 8 | Two hundred and sixty-six coaches with coaching experience in different sports (e.g., athletics, gymnastics, basketball, football etc.) consented to participation (45.9% coaches of athletes with II). Their average age was 40.88 (SD = 16.07, range 17 to 81 years old) and 58.6% of them were males |
|  |  | (b) Indicate number of participants with missing data for each variable of interest | - | No missing data |
|  |  | (c) *Cohort study*—Summarise follow-up time (eg, average and total amount) | - | - |
| Outcome data | 15* | *Cohort study*—Report numbers of outcome events or summary measures over time | *-* | *-* |
|  |  | *Case-control study—*Report numbers in each exposure category, or summary measures of exposure | *-* | *-* |
|  |  | *Cross-sectional study—*Report numbers of outcome events or summary measures | *10-12* | The rank MANOVA analysis showed that….. ….. Neither coaching style significantly predicted total performance progression in both groups. |
| Main results | 16 | (*a*) Give unadjusted estimates and, if applicable, confounder-adjusted estimates and their precision (eg, 95% confidence interval). Make clear which confounders were adjusted for and why they were included |  |  |
|  |  | (*b*) Report category boundaries when continuous variables were categorized |  |  |
|  |  | (*c*) If relevant, consider translating estimates of relative risk into absolute risk for a meaningful time period |  |  |

Continued on next page

| Other analyses | 17 | Report other analyses done—eg analyses of subgroups and interactions, and sensitivity analyses |  |  |
| --- | --- | --- | --- | --- |
| Discussion | | | | |
| Key results | 18 | Summarise key results with reference to study objectives | 13-17 | Results showed that perceived performance progression and controlled motivation were higher of athletes with II while perceived autonomous motivation was higher of athletes without II. No coaching style differences were found between the two groups. Additionally, a need-supportive coaching style negatively predicted amotivation, and a need-thwarting coaching style predicted lower autonomous motivation in athletes with II only. |
| Limitations | 19 | Discuss limitations of the study, taking into account sources of potential bias or imprecision. Discuss both direction and magnitude of any potential bias | 16-17 | A criticism of self-report measurements of sports performance development is that they could be affected by coaches’ bias towards athletes who have specific roles within the team |
| Interpretation | 20 | Give a cautious overall interpretation of results considering objectives, limitations, multiplicity of analyses, results from similar studies, and other relevant evidence | 13-17 | This study aimed to shed light on  …..to gain a better understanding of athletes’ progression and better support their long-term development in sports performance settings |
| Generalisability | 21 | Discuss the generalisability (external validity) of the study results | - | - |
| Other information | |  | | |
| Funding | 22 | Give the source of funding and the role of the funders for the present study and, if applicable, for the original study on which the present article is based | 17 | None |

*Give information separately for cases and controls in case-control studies and, if applicable, for exposed and unexposed groups in cohort and cross-sectional studies.

**Note:** An Explanation and Elaboration article discusses each checklist item and gives methodological background and published examples of transparent reporting. The STROBE checklist is best used in conjunction with this article (freely available on the Web sites of PLoS Medicine at http://www.plosmedicine.org/, Annals of Internal Medicine at http://www.annals.org/, and Epidemiology at http://www.epidem.com/). Information on the STROBE Initiative is available at www.strobe-statement.org.
